# A Family History of Stroke Is Associated with Increased Intima-Media Thickness in Young Ischemic Stroke - The Norwegian Stroke in the Young Study (NOR-SYS)

**DOI:** 10.1371/journal.pone.0159811

**Published:** 2016-08-09

**Authors:** Halvor Øygarden, Annette Fromm, Kristin Modalsli Sand, Christopher Elnan Kvistad, Geir Egil Eide, Lars Thomassen, Halvor Naess, Ulrike Waje-Andreassen

**Affiliations:** 1 Department of Clinical Medicine, University of Bergen, Bergen, Norway; 2 Department of Neurology, Haukeland University Hospital, Bergen, Norway; 3 Centre for Clinical Research, Haukeland University Hospital, Bergen, Norway; 4 Lifestyle Epidemiology Research Group, Department of Global Public Health and Primary Care, University of Bergen, Bergen, Norway; 5 Department of Biological and Medical Psychology, University of Bergen, Bergen, Norway; University of Milano, ITALY

## Abstract

**Background and Purpose:**

Positive family history (FH+) of cardiovascular disease (CVD) is a risk factor for own CVD. We aimed to analyze the effect of different types of FH (stroke, coronary heart disease (CHD), peripheral artery disease (PAD) on carotid intima-media thickness (cIMT) in young and middle-aged ischemic stroke patients.

**Methods:**

First-degree FH of CVD was assessed in ischemic stroke patients ≤ 60y using a standardized interview. Carotid ultrasound was performed and far wall cIMT in three carotid artery segments was registered, representing the common carotid (CCA-IMT), carotid bifurcation (BIF-IMT) and the internal carotid artery (ICA-IMT). Measurements were compared between FH+ and FH negative groups and stepwise backward regression analyses were performed to identify factors associated with increased cIMT.

**Results:**

During the study period 382 patients were enrolled, of which 262 (68%) were males and 233 (61%) reported FH of CVD. Regression analyses adjusting for risk factors revealed age as the most important predictor of cIMT in all segments. The association between FH+ and cIMT was modified by age (p = 0.014) and was significant only regarding ICA-IMT. FH+ was associated with increased ICA-IMT in patients aged < 45y (p = 0.001), but not in patients ≥ 45y (p = 0.083). The association with ICA-IMT was present for a FH of stroke (p = 0.034), but not a FH+ of CHD or PAD.

**Conclusions:**

FH of stroke is associated with higher ICA-IMT in young ischemic stroke patients. Subtyping of cardiovascular FH is important to investigate heredity in young ischemic stroke patients.

**Trial Registration:**

ClinicalTrials.gov NCT01597453

## Introduction

Family history (FH) of cardiovascular disease (CVD) is a risk factor for CVD.[1–3] Carotid intima media thickness (cIMT) is associated with CVD development, although the clinical value in predicting future vascular events is unclear.[4, 5] The 2013 update of the ACC/AHA guidelines on cardiovascular risk assessment withdrew the previous Class IIA recommendation to use cIMT measurements for cardiovascular risk stratification.[6] A recent meta-analysis found no association between cIMT progression measured by serial ultrasound measurements and future risk of CVD.[7] However, a recent study showed that increased cIMT is associated with risk factors for CVD and with first time stroke or myocardial infarction in patients < 45y.[8] Adding maximum internal carotid artery IMT (ICA-IMT) to a traditional risk stratification score has improved prediction of CVD events.[9] Increased cIMT is seen in children, adolescents and young adults with a history of premature parental coronary heart disease (CHD), suggesting an increased vulnerability to vascular risk factors.[10–13] A FH of premature CVD shows strong association with both CHD[14] and stroke[15] and the association between a FH of parental stroke <60y of age and increased ICA-IMT supports ICA-IMT as a good marker of hereditary stroke risk.[16] Increased ICA-IMT in ischemic stroke patients compared with controls and higher ICA-IMT in subjects with a FH of CVD have been reported in young patients.[17] Atherosclerosis is an inflammatory process that starts early in life, with a complex genetic and environmental chain of causality.[18–20] Increased IMT during childhood correlates well with the risk factor burden, and is considered an early manifestation of atherosclerosis.[21] ICA-IMT may be an easily accessible proxy to assess genetic risk in young ischemic stroke patients. We aimed to analyze associations between a FH of different CVD events and standardized segment cIMT measurements in young and middle-aged ischemic stroke patients included in the Norwegian Stroke in the Young Study (NOR-SYS).

## Materials and Methods

### Study population

All patients ≤ 60y with suspected stroke in the Bergen area with approximately 360,000 inhabitants, are admitted to the Stroke Unit at Haukeland University Hospital. From 1^rst^ September 2010 to 31^rst^ August 2015, 391consecutive patients aged 15 to 60y were prospectively included in NOR-SYS after documented acute ischemic stroke, 98.5% documented by magnetic resonance imaging (MRI) and the remaining by computed tomography (CT). The methods and rationale of NOR-SYS have been described in detail previously.[22] Three of the admitted patients died early from basilar occlusion and written consent with family members was not obtained. Four patients were adopted and had unknown biological parents and two patients were after inclusion found to have other disease than ischemic stroke, and were therefore excluded from this study. Young adults were defined < 45y, whereas patients aged 45 to 60y were defined as middle-aged.

### Family history

The FH of stroke, CHD and peripheral artery disease (PAD), was assessed in first-degree relatives (FDRs) using a face-to-face, standardized, questionnaire-based interview, as previously described.[23] The patient was interviewed within the first few days of admission and FH was considered positive (FH+) if the patient reported ≥ 1 FDR with CVD. The patient was designated FH negative (FH-) if no family members with CVD were reported. In 26 patients having aphasia or other communication difficulties a close relative was consulted as a secondary source of information during the interview, seven received help from one or both parents, 14 from a spouse, three from offspring and two from a sibling.

### Risk factor definitions

Patients were interviewed regarding working status and education and grouped accordingly. Diabetes mellitus (DM), hypertension (HT) and hypercholesterolemia were considered present if diagnosed before hospital admission and treated either by lifestyle changes and or medication. Years of treatment were available and calculated for DM and HT. Blood pressure (BP) was measured in both arms using appropriate cuff size after 15–30 minutes rest in supine position. These measurements were used to calculate mean systolic and diastolic BP. Serum glucose; low density lipoprotein (LDL), high density lipoprotein (HDL) and total cholesterol were measured. Patients were grouped as active smoker, previous smoker if cessation for at least one year, or never smoker. Degree of tobacco exposure was defined by pack-years, i.e. number of cigarette packs (20cig/pack) per day multiplied by number of years of active smoking. Body mass index (BMI) was calculated as weight in kilograms divided by height in meters squared. Waist-hip ratio (WHR) was calculated dividing waist circumference by hip circumference. Activity score was defined as an ordinal variable with range 0–5 and composed household activities, outdoor walks and active exercise. We defined inactivity/no physical activity of significance as 0, regular household activity and maintenance work as 1, outdoor walks 1-3h/week as 2, outdoor walks exceeding 3h/week as 3 and moderate to high intensity exercise 1-3h/week as 4, and moderate to high intensity exercise exceeding 3h/week as 5. Average weekly alcohol consumption of 0–3, 4–12, 13–20 or > 20 units per week was considered an ordinal variable defining never, low, moderate, high and excessive alcohol consumers, respectively.

### Carotid intima-media thickness

Carotid ultrasound was performed with a 3–9 MHz linear array transducer (Philips Medical Systems iU22, Bothell, WA, USA) and far wall IMT and/ or plaque was visualized using B-mode longitudinal plane scanning. The acquisition and measurement of cIMT have been described in detail previously.[22] Eight far-wall pictures were obtained at 4 standardized CCA angles, and four far-wall pictures were obtained at sites with maximum findings for the right and left BIF and ICA segments. We used Meijer’s Carotid Arc® for images at 180, 150, 120 and 90 degrees at the right carotid artery, at 180, 210, 240 and 270 degrees at the left carotid artery and saved all images at the end diastole, using electrocardiogram gating. The mean cIMT was analyzed offline using the QLAB automated IMT measurement plug-in (Philips, Bothell, WA) incorporating plaques if present at the pre-defined angle of image acquisition. Mean cIMT values from 10 mm measurements were registered from three carotid artery segments, defined as 20–10 mm, 10–0 mm proximal and 0–10 mm distal to the tip of the flow divider, representing the common carotid (CCA), carotid bifurcation (BIF) and internal carotid artery (ICA), respectively. All doctors involved in patient inclusion underwent an international training program with external accreditation before inclusion of patients. The highest cIMT values from either left or right side was used separately for CCA, BIF and ICA. The reliability of measurements within and between observers and equipment has been tested and reported previously.[17]

### Statistics

Descriptive statistics are reported using the mean and standard deviation (SD), median and interquartile range (IQR) or proportions with 95% confidence interval (CI). The chi square test was used for categorical data. Continuous data were analyzed by Student’s t-test or Mann–Whitney’s *U* test as appropriate. The cIMT-variables were highly skewed to the right and were therefore transformed. The transformation of cIMT by 1/(square root of cIMT) gave distributions and residual distributions resembling a normal distribution and was therefore chosen as the most appropriate conversion for linear regression analyses. Of note, this inverse square root transformation means apparent negative associations must be interpreted as positive.

Multiple linear regression analysis was performed to test associations with cIMT. Results were reported as regression coefficients (coef.) and standard errors (SE). All regression analyses were performed as stepwise backwards procedures to identify factors associated with increased or reduced cIMT. The first step regression model included age, sex, FH of CVD, WHR, BMI, smoking status, pack-years of smoking, activity score, alcohol consumption score, hypercholesterolemia, HT, years of HT treatment, DM, years of DM treatment, mean systolic BP, mean diastolic BP, number of siblings, educational category, working status, s-glucose, s-HDL-C, s-LDL-C and s-total cholesterol. The least significant variable was removed in each step of the procedure until only significant variables remained (p ≤ 0.05). Age and sex were forced into all models. Finally, putative interactions with age and sex were added to the model and tested. If IMT measurements were missing due to severe atherosclerosis documented by autopsy, angiographic imaging or carotid surgery before ultrasound examinations the missing data points in regression analyses were treated with 90^th^ percentile high value imputation, robustness of this approach was tested by repeating the regression analyses using list-wise deletion and imputation of low and mean values. Missing measurements due to anatomical variations were treated by mean imputation and robustness was tested with a similar approach. Robust standard errors were used for calculation.

The level of significance was set at 0.05. Stata 13.1 (StataCorp, College Station, TX) was used for all analyses.

### Ethics

All patients or legal guardians signed a written informed consent. NOR-SYS is approved by the Regional Ethics Committee of Western Norway, and is conducted in accordance with the Declaration of Helsinki.

## Results

### Demographics

Over a period of 5 years 382 ischemic stroke patients were enrolled in this study, of which 262 (68%) were males and 233 (61%) reported FH of CVD in ≥ 1 FDR. A FH of stroke was reported by 128 (33.5%), while 167 (43.7%) and 26 (6.8%) reported a FH of CHD and PAD, respectively. Cigarette smoking (p < 0.001) and HT (p = 0.001) were more common in the FH+ group. The FH+ patients had lower mean activity score (p = 0.004) and were less educated with 33% graduating from college or university compared to 47% of FH- patients (p = 0.016). FH+ patients had higher WHR, BP, LDL-C and total cholesterol (all p ≤ 0.005). In 10 (2.6%) patients anamnestic values were used to calculate BMI. All seven patients with missing measurements due to atherosclerotic occlusions were middle-aged and six of them had FH+ (p = 0.859). The demographic data are presented stratified between young and middle-aged due to the age-related discrepancy in FH+ and FH- in these groups, respectively (p < 0.001 and p = 0.400; Table 1).

**Table 1:** Demographic data of 382 young and middle-aged patients included in—the Norwegian Stroke in the Young Study (NOR-SYS)—in Bergen, Norway 2010–2015.

		Age 15-44y (N = 94)			Age 45-60y (N = 288)		
Variables		FH+ (n = 25, 27%)	FH- (n = 69, 73%)	P	FH+ (n = 208, 72%)	FH- (n = 80, 28%)	P
Age in years		39.7 (4.6)	33.7 (8.4)	0.001^†^	54.2 (4.5)	53.8 (4.3)	0.400^†^
Male sex, *n* (%)		11 (44.0)	42 (60.9)	0.145*	147 (70.7)	62 (77.5)	0.245*
Education, *n* (%)				0.017*			0.925*
	Basic school	7 (28.0)	8 (11.8)		61 (29.9)	22 (27.9)	
	High school	11 (44.0)	19 (28.0)		75 (36.8)	29 (36.7)	
	College/University	7 (28.0)	41 (60.3)		68 (33.3)	28 (35.4)	
Working status ^a^, *n* (%)				0.513*			0.982*
	Full-time	18 (72.0)	55 (82.1)		136 (66.0)	52 (66.7)	
	Part-time	3 (12.0)	4 (6.0)		23 (11.2)	9 (11.5)	
	Unemployed	4 (16.0)	8 (11.0)		47 (22.8)	17 (21.8)	
Cigarette smoking, *n* (%)				0.007*			0.192*
	Never	5 (20.0)	39 (56.5)		47 (22.6)	26 (32.9)	
	Former	6 (24.0)	10 (14.5)		57 (27.4)	20 (25.3)	
	Active	14 (56.0)	20 (29.0)		104 (50.0)	33 (41.8)	
High/excessive alcohol, *n* (%)		2 (8.0)	4 (5.8)	0.630*	22 (10.6)	9 (11.3)	0.557*
Activity score		2.7 (1.4)	3.4 (1.6)	0.023^†^	2.6 (1.4)	2.7 (1.5)	0.466^†^
CVD events, *n* (%)		1 (4.0)	6 (8.7)	0.444	55 (26.4)	12 (15.0)	0.040
BP-lowering therapy, *n* (%)		3 (12.0)	15 (21.7)	0.289*	97 (48.0)	26 (32.9)	0.022*
DM therapy, *n* (%)		1 (4.0)	3 (4.4)	0.941*	21 (10.2)	9 (11.2)	0.794*
Lipid-lowering therapy, *n* (%)		0 (0.0)	1 (1.4)	0.545*	37 (17.8)	10 (12.5)	0.277*
Systolic BP, mmHg		131 (14)	131 (17)	0.563^†^	145 (19)	141 (15)	0.102^†^
Diastolic BP, mmHg		81 (11)	81 (14)	0.615^†^	89 (13)	86 (11)	0.036^†^
Body-mass index, kg/m^2^		27.8 (6.4)	26.9 (6.4)	0.317^†^	27.7 (4.5)	27.0 (4.7)	0.390^†^
Waist-hip ratio		0.88 (0.07)	0.87 (0.1)	0.470^†^	0.94 (0.08)	0.93 (0.09)	0.319^†^
Total cholesterol, mmol/L		5.49 (1.5)	4.97 (1.1)	0.101^†^	5.50 (1.2)	5.28 (1.2)	0.254^†^
LDL-C, mmol/L		3.67 (1.3)	3.08 (1.0)	0.028^†^	3.70 (1.1)	3.44 (1.1)	0.110^†^
HDL-C, mmol/L		1.34 (0.4)	1.51 (0.6)	0.309^†^	1.32 (0.4)	1.33 (0.5)	0.956^†^
Glucose, mmol/L		5.4 (0.6)	5.7 (1.0)	0.139^†^	6.7 (2.4)	7.3 (3.3)	0.253^†^
Number of siblings, *median (IQR)*		1 (1–2)	2 (1–3)	0.197^†^	2 (2–4)	2 (1–3)	0.024^†^
Number of children, *median (IQR)*		1 (0–3)	1 (0–2)	0.452^†^	2 (1–3)	2 (2–3)	0.460^†^

Values are given as mean with standard deviation unless otherwise stated.

*Abbreviations*: *n* (%): absolute number of patients and percentage within group; CVD: cardiovascular disease; FH: Family history, i.e. FH+: at least one first-degree relative with CVD; DM: diabetes mellitus; BP: blood pressure; LDL-C: low-density lipoprotein cholesterol; HDL-C: high-density lipoprotein cholesterol; IQR: interquartile range.

^a^ Full-time working status, includes students/pupils; part-time, includes stay at home parents and part-time benefit recipients if also working; unemployed includes full benefit recipients and 3 young and 10 middle-aged patients on long term (> 6months) sick-leave.

* Chi square test.

† Mann–Whitney *U* test.

The young FH+ group had more deceased parents than the FH- with 9 (36%) deceased fathers and 3 (12.5%) mothers compared with 9 (13%) and 3 (4.4%) in the FH- group (p = 0.014 and 0.161). No difference in numbers of deceased parents was observed in the middle-aged group.

### IMT measurements and predictors of increased IMT

One patient (0.2%) had unsuccessful BIF-IMT, whereas six (1.5%) had unsuccessful ICA-IMT measurements on both sides. One patient had no successful ultrasound IMT measurements due to technical difficulties. Due to the large impact of age on IMT and FH and the skewed age distribution, IMT data are shown stratified by FH and age (Table 2). In young patients the highest mean ICA-IMT was 0.265 mm (49%) higher and BIF-IMT measurements were 0.17 mm (24%) higher in FH+ compared with FH- patients (p < 0.001 and p = 0.002). No significant difference was found regarding CCA or in any segment in middle-aged patients.

**Table 2:** Ultrasound measurements of the segmental carotid intima-media thickness in 382 young and middle-aged patients included in the Stroke in the Young Study (NOR-SYS) in Bergen, Norway 2010–2015.

	Age 15-44y			Age 45-60y		
Variables	FH+ (n = 25	FH-(n = 69)	P	FH+ (n = 208)	FH- (n = 80)	P
Mean IMT right CCA, mm	0.611 (0.13)	0.587 (0.12)	0.342	0.834 (0.25)	0.774 (0.19)	0.094
Mean IMT right BIF, mm	0.781 (0.38)	0.642 (0.26)	0.021	1.200 (0.75)	1.083 (0.62)	0.183
Mean IMT right ICA, mm	0.762 (0.44)	0.519 (0.19)	0.002	0.989 (0.75)	1.115 (1.02)	0.763
Mean IMT left CCA, mm	0.871 (1.21)	0.586 (0.12)	0.033	0.855 (0.28)	0.815 (0.27)	0.182
Mean IMT left BIF, mm	0.778 (0.27)	0.622 (0.23)	< 0.001	1.093 (0.57)	1.101 (0.52)	0.851
Mean IMT left ICA, mm	0.659 (0.35)	0.475 (0.11)	< 0.001	0.840 (0.57)	0.976 (0.85)	0.160
Highest mean IMT CCA, mm	0.886 (1.21)	0.623 (0.12)	0.126	0.922 (0.30)	0.858 (0.27)	0.117
Highest mean IMT BIF, mm	0.864 (0.36)	0.694 (0.29)	0.002	1.361 (0.80)	1.318 (0.67)	0.871
Highest mean IMT ICA, mm	0.806 (0.42)	0.541 (0.18)	< 0.001	1.075 (0.75)	1.281 (1.09)	0.327

Values are given as mean with standard deviation unless otherwise stated.

*Abbreviations*: FH: family history; IMT: intima-media thickness; CCA: common carotid artery; BIF: carotid bifurcation; ICA: internal carotid artery; SD: standard deviation; P: p-value of comparison between FH+ and FH- groups calculated by Mann–Whitney’s *U* test.

The variables associated with increased CCA-IMT after backwards selection procedure were age, male sex, HT, mean systolic BP, pack-years of smoking and LDL-cholesterol (all p < 0.008). The variables associated with increased BIF-IMT were high age, previous CVD, mean systolic BP, years of DM, active smoking, increased LDL-cholesterol (all p < 0.004) and lower HDL-cholesterol (p < 0.001). The variables associated with increased ICA-IMT included FH, age, mean systolic BP, pack years of smoking, years of DM and the interaction term between FH and age (FH×age) (all p ≤ 0.013). The FH×age term showed effect modification of the association between FH and ICA-IMT by age with increasing age attenuating the effect of a positive FH on IMT (p = 0.006). No interactions between age and other risk factors were significant.

**Fig 1:**
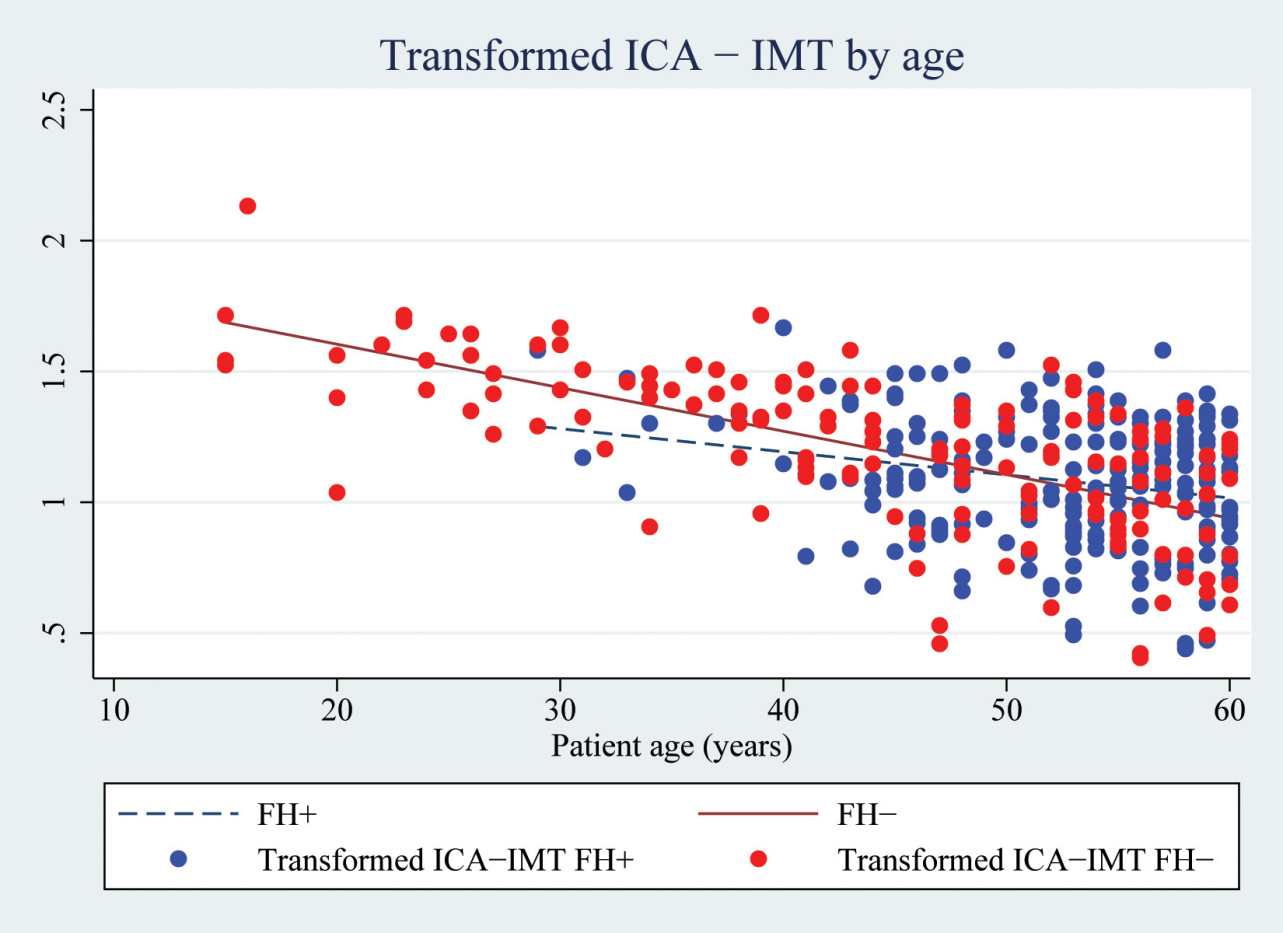
Transformed ICA-IMT by age. The distribution of the transformed ICA-IMT (1/√ICA-IMT) is plotted by age in 382 young and middle-aged ischemic stroke patients included in the Norwegian Stroke in the Young Study (NOR-SYS) in Bergen, Norway 2010–2015. Lines of best fit for FH+ and FH- patients are superimposed on the plot. There is a strong negative correlation between the 1/√ICA-IMT and age (Pearson’s r = -0.514; p < 0.001). The large divergence of the lines at young age indicates the predictive value of a positive FH on the transformed ICA-IMT is high at young age and the lines converging with progressing age shows the association attenuates with increasing age.

FH is associated with higher ICA-IMT in patients < 45y. The effect of FH+ on ICA-IMT attenuates with increasing age and after the age of 50y a converse association seems apparent, as shown in Fig 1.

Splitting the FH in subdomains to analyze if a FH of stroke, CHD or PAD differed with regard to impact on ICA-IMT showed only a FH of stroke was significantly associated with higher ICA-IMT in stroke patients (p = 0.011; Table 3). No imputation method for missing data altered the main results. However, imputing expected high ICA-IMT values in patients with documented ICA atherosclerosis strengthened the main association between FH of stroke and ICA-IMT, whereas imputing low values attenuated the association.

**Table 3:** Multiple linear regression analysis displaying factors associated with the transformed internal carotid artery-intima media thickness (1/√ICA-IMT) in 382 ischemic stroke patients aged 15-60y included in the Norwegian Stroke in Young Study (NOR-SYS) Bergen, Norway 2010–2015.

Variable^a^		Coef.	95%CI	P–value
Age		-0.013	-0.017 –-0.010	< 0.001
Sex		0.432	-0.032–0.074	0.432
FH of CVD	No FH	0		
	FH of stroke	-0.528	-0.936 –-0.121	0.011
	FH of CHD	-0.383	-0.786–0.019	0.062
	FH of PAD	1.897	-0.608–4.403	0.137
	FH of several	-0.044	-0.548–0.460	0.863
FH type X age ^b^	No FH	0		
	FH of stroke	0.0117	0.003–0.019	0.004
	FH of CHD	0.0075	0.000–0.015	0.065
	FH of PAD	-0.0329	-0.075–0.009	0.132
	FH of several	0.0010	-0.008–0.010	0.828
Body-mass index		-0.005	-0.010 –-0.001	0.025
Mean blood pressure		-0.002	-0.003 –-0.001	0.020
Years of diabetes mellitus		-0.009	-0.015 –-0.004	0.001
Pack years of smoking		-0.003	-0.004–0.001	< 0.001

*Abbreviations*: Coef.: regression coefficient; 95%CI: 95% confidence interval; FH: Family history; CVD: cardio-vascular disease; CHD: coronary heart disease; PAD: peripheral artery disease.

^a^ Remaining variables from backwards stepwise selection procedure with first step including age, sex, FH of CVD, waist-hip ratio, body mass index, smoking status, pack-years of smoking, activity score, alcohol consumption score, hypercholesterolemia, hypertension, years of hypertension treatment, diabetes mellitus, years of diabetes mellitus treatment, mean systolic blood pressure, mean diastolic blood pressure, number of siblings, working status, educational category, s-glucose, s-HDL-C, s-LDL-C and s-total cholesterol.

^b^ Interaction term describing effect modification by age on the association between a FH of stroke and ICA-IMT.

## Discussion

We found FH+ of CVD associated with increased ICA-IMT in young ischemic stroke patients. The association was present only for a FH of stroke, although a FH of CHD was borderline significant. This expands on the recent finding that common carotid IMT measurements in patients < 45y improves CVD event prediction.[8] The increased ICA-IMT in young FH+ patients indicate that genetic factors seem to have more impact on ICA-IMT and risk of CVD in young compared with middle-aged patients. Given the association between ICA-IMT and FH is driven by a FH of stroke, the hereditary factor for increased ICA-IMT seems to be stroke specific. We found a significant association between age and IMT in all carotid artery segments, and in addition we found the IMT of the BIF and the CCA are associated with traditional and modifiable risk factors such as smoking, DM, BP and cholesterol levels. These findings are in line with previous studies, although varying impact of risk factors on IMT has been reported.[24, 25] Several of these risk factors such as BP[26] and cholesterol levels[27] are shown to have a strong hereditary component themselves, and this may influence the impact of FH+ in our regression analyses. Regarding ICA-IMT in the middle-aged group, there was a pattern with increased impact of traditional risk factors whereas the impact of genetics represented by FH+ was lower as compared with the young group. This may contribute to explain why cIMT has less impact on risk prediction in older patients.[5–7] In addition, previous CVD was more common in the middle-aged FH+ patients with 26% vs. 15% in the FH- group. This difference may be prone to bias by age, due to the large impact of age on both prior CVD in patients and FH status of patients. The lack of association between a FH of CVD and ICA-IMT in middle-aged patients may be explained by our definition of FH of CVD, with no age limit imposed on the FH. With this definition of FH we show the middle-aged patients have a much higher prevalence of FH+ than the young, probably to a large degree caused by the higher age of the relatives and thus the higher prevalence of CVD with progressing age. Increasing prevalence of FH+ with increasing age has recently been reported from another large European cohort of young ischemic stroke patients.[28] With no age-limits on the FH the increased FH of CVD prevalence in the middle-aged may be largely caused by parental events at high age thereby diluting the possible effect of a FH of premature CVD in the middle-aged. [1]

The Mannheim consensus recommends cIMT measurements in a distal CCA region free of plaque and with an easily discernable IMT double-line pattern, ensuring high reproducibility and comparable results between populations.[29] An approach using one of three carotid segments with the fastest progressing IMT, allowing incorporation of plaques in the measurement showed significant association with subsequent vascular events.[30] This supports the feasibility of a maximum pathology approach if cIMT is used for risk prediction. There is evidence that genetic factors explain a large part of phenotypic cIMT variation, with studies reporting 30–66% of CCA-IMT and even up to 75% of ICA-IMT variation accounted for by genes.[25, 31–33] This suggests ICA-IMT as an appropriate measure in investigations of genetic stroke risk.[15, 25] Our finding of a strong association between a FH of stroke and increased ICA-IMT supports this. Adjusting the strategy for future genetic analyses in stroke seems imperative given that even large genetic consortia have only uncovered a few genetic variants with low explanatory effect on ischemic stroke.[34] A thorough standardized clinical work-up and phenotypic stratification of stroke patients seems a promising approach to enable collaboration for future genetic studies.[35]

The main strengths of our study are the detailed and standardized multi-angle assessment of pre-defined carotid artery segments and the detailed FH divided in components of stroke, CHD and PAD. Further strengths include the prospective and consecutive inclusion of patients, the strict inclusion criteria with mandatory verification of acute cerebral infarction by a high rate of MRI and extensive risk factor evaluation. There are some limitations to our study. Firstly, parent age at CVD event was not systematically ascertained, using premature FH could potentially strengthen the associations between FH and cIMT.[12] Secondly, self-reported FH data carries a risk of recall bias. However, reliability of the patient-reported FH from this cohort has previously been tested against the reports from living parents resulting in adequate concordance.[36]

The present paper shows an association between a FH of stroke and increased ICA-IMT in young ischemic stroke patients. Most of the genetic risk in vascular disease is polygenic, with the exception of monogenic disorders such as CADASIL, identifying homogenous phenotypes allows a more targeted approach in the search for genetic variants associated with increased stroke risk.[35, 37] The use of ultrasound ICA-IMT measurements in young patients may assist in identification of individuals with a genetic CVD component, thereby allowing targeted advice on lifestyle alterations such as exercise, weight loss and smoking cessation to potentially reduce future CVD related disability and mortality. Our results suggest future guidelines for the assessment of stroke risk should consider recommendations of ICA-IMT measurements in young adults.

## Conclusions

The present paper shows association between a family history of stroke and increased intima media thickness of the internal carotid artery in young ischemic stroke patients. This supports the influence of a genetic component in young stroke and suggests that measurements of intima media thickness can identify young patients with a hereditary risk for stroke. Genetic research regarding atherosclerosis in stroke should target young individuals with a high likelihood of genetic disease components. Additionally, identifying individuals with a hereditary risk enables targeted advice on lifestyle alterations such as exercise, weight loss and smoking cessation to reduce future disability and mortality caused by cardiovascular disease.

## References

[1] Lloyd-Jones DM, Nam BH, D'Agostino RB Sr., Levy D, Murabito JM, Wang TJ, et al Parental cardiovascular disease as a risk factor for cardiovascular disease in middle-aged adults: a prospective study of parents and offspring. JAMA: the journal of the American Medical Association. 2004;291(18):2204–11. Epub 2004/05/13. 10.1001/jama.291.18.2204 . 15138242

[2] Banerjee A. A review of family history of cardiovascular disease: risk factor and research tool. Int J Clin Pract. 2012;66(6):536–43. Epub 2012/05/23. 10.1111/j.1742-1241.2012.02908.x . 22607505

[3] Pandey AK, Pandey S, Blaha MJ, Agatston A, Feldman T, Ozner M, et al Family history of coronary heart disease and markers of subclinical cardiovascular disease: where do we stand? Atherosclerosis. 2013;228(2):285–94. Epub 2013/04/13. 10.1016/j.atherosclerosis.2013.02.016 . 23578356

[4] Lorenz MW, Markus HS, Bots ML, Rosvall M, Sitzer M. Prediction of clinical cardiovascular events with carotid intima-media thickness: a systematic review and meta-analysis. Circulation. 2007;115(4):459–67. Epub 2007/01/24. 10.1161/CIRCULATIONAHA.106.628875 . 17242284

[5] Den Ruijter HM, Peters SA, Anderson TJ, Britton AR, Dekker JM, Eijkemans MJ, et al Common carotid intima-media thickness measurements in cardiovascular risk prediction: a meta-analysis. JAMA: the journal of the American Medical Association. 2012;308(8):796–803. Epub 2012/08/23. 10.1001/jama.2012.9630 . 22910757

[6] Goff DC Jr., Lloyd-Jones DM, Bennett G, Coady S, D'Agostino RB Sr., Gibbons R, et al 2013 ACC/AHA guideline on the assessment of cardiovascular risk: a report of the American College of Cardiology/American Heart Association Task Force on Practice Guidelines. Journal of the American College of Cardiology. 2014;63(25 Pt B):2935–59. Epub 2013/11/19. 10.1016/j.jacc.2013.11.005 . 24239921 PMC4700825

[7] Lorenz MW, Polak JF, Kavousi M, Mathiesen EB, Volzke H, Tuomainen TP, et al Carotid intima-media thickness progression to predict cardiovascular events in the general population (the PROG-IMT collaborative project): a meta-analysis of individual participant data. Lancet. 2012;379(9831):2053–62. Epub 2012/05/01. 10.1016/S0140-6736(12)60441-3 22541275 PMC3918517

[8] Eikendal AL, Groenewegen KA, Anderson TJ, Britton AR, Engstrom G, Evans GW, et al Common Carotid Intima-Media Thickness Relates to Cardiovascular Events in Adults Aged <45 Years. Hypertension. 2015 Epub 2015/01/28. 10.1161/HYPERTENSIONAHA.114.04658 . 25624341

[9] Polak JF, Pencina MJ, Pencina KM, O'Donnell CJ, Wolf PA, D'Agostino RB Sr,. Carotid-wall intima-media thickness and cardiovascular events. The New England journal of medicine. 2011;365(3):213–21. Epub 2011/07/22. 10.1056/NEJMoa1012592 21774709 PMC3153949

[10] Cuomo S, Guarini P, Gaeta G, De Michele M, Boeri F, Dorn J, et al Increased carotid intima-media thickness in children-adolescents, and young adults with a parental history of premature myocardial infarction. European heart journal. 2002;23(17):1345–50. Epub 2002/08/23. . 12191745 10.1053/euhj.2001.3111

[11] Gaeta G, De Michele M, Cuomo S, Guarini P, Foglia MC, Bond MG, et al Arterial abnormalities in the offspring of patients with premature myocardial infarction. The New England journal of medicine. 2000;343(12):840–6. Epub 2000/09/21. 10.1056/NEJM200009213431203 . 10995863

[12] Wang TJ, Nam B-H, D’Agostino RB, Wolf PA, Lloyd-Jones DM, MacRae CA, et al Carotid Intima-Media Thickness Is Associated With Premature Parental Coronary Heart Disease The Framingham Heart Study. Circulation. 2003;108(5):572–6. 12874190 10.1161/01.CIR.0000081764.35431.DE

[13] Juonala M, Viikari JS, Räsänen L, Helenius H, Pietikäinen M, Raitakari OT. Young Adults With Family History of Coronary Heart Disease Have Increased Arterial Vulnerability to Metabolic Risk Factors The Cardiovascular Risk in Young Finns Study. Arteriosclerosis, thrombosis, and vascular biology. 2006;26(6):1376–82. 16614318 10.1161/01.ATV.0000222012.56447.00

[14] Chow CK, Islam S, Bautista L, Rumboldt Z, Yusufali A, Xie C, et al Parental history and myocardial infarction risk across the world: the INTERHEART Study. Journal of the American College of Cardiology. 2011;57(5):619–27. Epub 2011/01/29. 10.1016/j.jacc.2010.07.054 . 21272754

[15] Jerrard-Dunne P, Cloud G, Hassan A, Markus HS. Evaluating the genetic component of ischemic stroke subtypes: a family history study. Stroke; a journal of cerebral circulation. 2003;34(6):1364–9. Epub 2003/04/26. 10.1161/01.STR.0000069723.17984.FD . 12714707

[16] Ziegelbauer K, Schaefer C, Steinmetz H, Sitzer M, Lorenz MW. Clinical usefulness of carotid ultrasound to improve stroke risk assessment: ten-year results from the Carotid Atherosclerosis Progression Study (CAPS). European journal of preventive cardiology. 2013;20(5):837–43. Epub 2012/05/24. 10.1177/2047487312449589 . 22617119

[17] Fromm A, Haaland OA, Naess H, Thomassen L, Waje-Andreassen U. Risk factors and their impact on carotid intima-media thickness in young and middle-aged ischemic stroke patients and controls: the Norwegian Stroke in the Young Study. BMC research notes. 2014;7:176 Epub 2014/03/29. 10.1186/1756-0500-7-176 24669965 PMC3986875

[18] McGill HC, McMahan CA, Herderick EE, Malcom GT, Tracy RE, Strong JP. Origin of atherosclerosis in childhood and adolescence. The American journal of clinical nutrition. 2000;72(5):1307s–15s. 11063473 10.1093/ajcn/72.5.1307s

[19] McGill HC, McMahan CA, Gidding SS. Preventing heart disease in the 21st century implications of the pathobiological determinants of atherosclerosis in youth (PDAY) study. Circulation. 2008;117(9):1216–27. 10.1161/CIRCULATIONAHA.107.717033 18316498

[20] Mozaffarian D, Benjamin EJ, Go AS, Arnett DK, Blaha MJ, Cushman M, et al Heart disease and stroke statistics—2015 update: a report from the American Heart Association. Circulation. 2015;131(4):e29–322. Epub 2014/12/19. 10.1161/CIR.0000000000000152 . 25520374

[21] Juonala M, Magnussen CG, Venn A, Dwyer T, Burns TL, Davis PH, et al Influence of age on associations between childhood risk factors and carotid intima-media thickness in adulthood: the Cardiovascular Risk in Young Finns Study, the Childhood Determinants of Adult Health Study, the Bogalusa Heart Study, and the Muscatine Study for the International Childhood Cardiovascular Cohort (i3C) Consortium. Circulation. 2010;122(24):2514–20. Epub 2010/12/04. 10.1161/CIRCULATIONAHA.110.966465 . 21126976

[22] Fromm A, Thomassen L, Naess H, Meijer R, Eide GE, Krakenes J, et al The Norwegian Stroke in the Young Study (NOR-SYS): Rationale and design. BMC neurology. 2013;13(1):89 Epub 2013/07/20. 10.1186/1471-2377-13-89 23865483 PMC3721997

[23] Øygarden H, Fromm A, Sand KM, Eide GE, Thomassen L, Naess H, et al Stroke patients’ knowledge about cardiovascular family history-the Norwegian stroke in the Young study (NOR-SYS). BMC neurology. 2015;(1):30. 25884546 10.1186/s12883-015-0276-6 PMC4359475

[24] Chambless LE, Heiss G, Folsom AR, Rosamond W, Szklo M, Sharrett AR, et al Association of coronary heart disease incidence with carotid arterial wall thickness and major risk factors: the Atherosclerosis Risk in Communities (ARIC) Study, 1987–1993. American journal of epidemiology. 1997;146(6):483–94. Epub 1997/09/18. . 9290509 10.1093/oxfordjournals.aje.a009302

[25] Rundek T, Blanton SH, Bartels S, Dong C, Raval A, Demmer RT, et al Traditional risk factors are not major contributors to the variance in carotid intima-media thickness. Stroke; a journal of cerebral circulation. 2013;44(8):2101–8. Epub 2013/05/25. 10.1161/STROKEAHA.111.000745 23704105 PMC3738011

[26] Levy D, Ehret GB, Rice K, Verwoert GC, Launer LJ, Dehghan A, et al Genome-wide association study of blood pressure and hypertension. Nature genetics. 2009;41(6):677–87. Epub 2009/05/12. 10.1038/ng.384 19430479 PMC2998712

[27] Kathiresan S, Manning AK, Demissie S, D'agostino RB, Surti A, Guiducci C, et al A genome-wide association study for blood lipid phenotypes in the Framingham Heart Study. BMC medical genetics. 2007;8(Suppl 1):S17 17903299 10.1186/1471-2350-8-S1-S17 PMC1995614

[28] Thijs V, Grittner U, Dichgans M, Enzinger C, Fazekas F, Giese A-K, et al Family History in Young Patients With Stroke. Stroke; a journal of cerebral circulation. 2015;46(7):1975–8. 10.1161/STROKEAHA.115.009341 26038521

[29] Touboul PJ, Hennerici MG, Meairs S, Adams H, Amarenco P, Bornstein N, et al Mannheim carotid intima-media thickness and plaque consensus (2004-2006-2011). An update on behalf of the advisory board of the 3rd, 4th and 5th watching the risk symposia, at the 13th, 15th and 20th European Stroke Conferences, Mannheim, Germany, 2004, Brussels, Belgium, 2006, and Hamburg, Germany, 2011. Cerebrovascular diseases. 2012;34(4):290–6. Epub 2012/11/07. 10.1159/000343145 23128470 PMC3760791

[30] Baldassarre D, Veglia F, Hamsten A, Humphries SE, Rauramaa R, de Faire U, et al Progression of carotid intima-media thickness as predictor of vascular events: results from the IMPROVE study. Arterioscler Thromb Vasc Biol. 2013;33(9):2273–9. Epub 2013/07/05. 10.1161/ATVBAHA.113.301844 . 23825364

[31] Duggirala R, Gonzalez Villalpando C, O'Leary DH, Stern MP, Blangero J. Genetic basis of variation in carotid artery wall thickness. Stroke; a journal of cerebral circulation. 1996;27(5):833–7. Epub 1996/05/01. . 8623101 10.1161/01.str.27.5.833

[32] Zannad F, Visvikis S, Gueguen R, Sass C, Chapet O, Herbeth B, et al Genetics strongly determines the wall thickness of the left and right carotid arteries. Human genetics. 1998;103(2):183–8. Epub 1998/10/06. . 9760203 10.1007/s004390050804

[33] Sacco RL, Blanton SH, Slifer S, Beecham A, Glover K, Gardener H, et al Heritability and linkage analysis for carotid intima-media thickness: the family study of stroke risk and carotid atherosclerosis. Stroke; a journal of cerebral circulation. 2009;40(7):2307–12. Epub 2009/06/06. 10.1161/STROKEAHA.109.554121 19498180 PMC2737512

[34] International Stroke Genetics C, Wellcome Trust Case Control C, Bellenguez C, Bevan S, Gschwendtner A, Spencer CC, et al Genome-wide association study identifies a variant in HDAC9 associated with large vessel ischemic stroke. Nature genetics. 2012;44(3):328–33. Epub 2012/02/07. 10.1038/ng.1081 22306652 PMC3303115

[35] Majersik JJ, Cole JW, Golledge J, Rost NS, Chan Y-FY, Gurol ME, et al Recommendations From the International Stroke Genetics Consortium, Part 1 Standardized Phenotypic Data Collection. Stroke; a journal of cerebral circulation. 2015;46(1):279–84. 10.1161/STROKEAHA.114.006839 25492903 PMC4465378

[36] Oygarden H, Fromm A, Sand KM, Eide GE, Thomassen L, Naess H, et al Can the cardiovascular family history reported by our patients be trusted? The Norwegian Stroke in the Young Study. European journal of neurology: the official journal of the European Federation of Neurological Societies. 2015 Epub 2015/08/22. 10.1111/ene.12824 . 26293608 PMC5049640

[37] Lusis AJ. Genetics of atherosclerosis. Trends in Genetics. 2012;28(6):267–75. 10.1016/j.tig.2012.03.001 22480919 PMC3362664

